# First year medical student performance on weekly team-based learning exercises in an infectious diseases course: insights from top performers and struggling students

**DOI:** 10.1186/s12909-019-1608-9

**Published:** 2019-06-03

**Authors:** Gonzalo A. Carrasco, Kathryn C. Behling, Osvaldo J. Lopez

**Affiliations:** 1 Department of Biomedical Sciences, Cooper Medical School of Rowan University, 401 S. Broadway, Camden, NJ 08103 USA; 2 Department of Medical Sciences, Hackensack Meridian School of Medicine at Seton Hall University, 340 Kingsland Street, Nutley, NJ 07110 USA

**Keywords:** Active learning, Team-based learning, Individual readiness assurance test, Performance, Self-directed learning, Pre-class preparation

## Abstract

**Background:**

In Team-Based Learning (TBL) preparation of relevant coursework during self-directed learning time is evaluated by the individual readiness assurance test (iRAT). We recently reported that student performance on iRATs is strongly correlated with final examination scores in an infectious diseases (ID) course. We now investigated how student preparation for each individual iRAT exercise relates to course performance.

**Methods:**

Two-hundred and sixty medical students were enrolled in this three-year study. Student TBL iRAT scores were collected and correlated with final examination scores using Kruskal-Wallis One-Way ANOVA and Newman-Keul’s statistical methods.

**Results:**

Students performing in the upper and middle 33rd percentile on the final examination showed highly significant (*p* < 0.01) weekly improvements in their iRAT scores. However, students performing in the lower 33rd percentile did not show improvement in their iRAT scores until the last week of the course. Although there was a highly significant correlation between final examination and iRAT scores amongst all students participating in the study, this correlation was stronger in students performing in the lower 33rd percentile.

**Conclusions:**

Our data suggest that students who do not consistently prepare for TBL, as evidenced by low iRAT scores, exhibit poorer performance on the final examination. This lack of preparation likely interferes with the efficacy of this learning method. iRAT scores can also be used for early identification of struggling students in need of additional supports. Additionally, changes in TBL incentive structure may provide more tangible rewards for pre-class preparation in particular for struggling students.

## Background

Educational strategies that promote active learning, such as Problem-Based Learning **(**PBL), Case-Based Learning (CBL), and Team-Based Learning (TBL), are essential to affording students the necessary tools for lifelong learning. Several studies have provided strong evidence that TBL exercises improve educational outcomes for students in multiple disciplines [1–3] and settings [4, 5]. We and others have also reported the beneficial effects of TBL in medical education [1, 6–9], where it improves the learning experience [10] and helps students to better understand complex subjects [1]. L. Michelsen, who developed TBL to address concerns regarding student engagement in a large class of undergraduate students, has suggested that properly formed teams, student accountability, frequent and timely feedback to students, and team assignments that promote learning and team development, are key to the success of the TBL experience [11, 12].

In the first phase of TBL [13], learners study independently outside of class to master identified objectives. In the second phase, mastery of relevant course material prepared during self-directed learning time is evaluated at the beginning of the TBL activity in a quantitative fashion using a graded multiple-choice quiz, the individual readiness assessment test (iRAT). In this phase, learners also work together in teams of 5–6 students to complete the group readiness assurance test (gRAT), which is composed of the same quiz questions as the iRAT. At the end of the gRAT, it is expected that students will achieve mastery of the target learning objectives through peer-to-peer teaching [11, 12]. We recently reported that implementation of TBL exercises in an Infectious Disease (ID) course at our institution resulted in improved performance on final examinations, in particular for more poorly performing students [6–8].

Several groups have studied the effects of incentive structure and grading of TBL exercises on the efficacy of this method [14–16]. Some studies have suggested that ungraded TBLs would be more student friendly and would support the active learning environment while reinforcing teamwork skills [15]. However, we found that at our institution, iRAT scores are higher and are also better predictors of course success when TBL exercises are graded as opposed to ungraded [7]. Furthermore, we recently reported that in graded TBL exercises, iRAT performance, a surrogate for individual student preparation for TBL exercises, correlated with final examination scores [6]. Accordingly, a recent study suggests that medical students see graded TBL exercises as the optimal teaching strategy over other active learning activities, such as PBL, because the structure and format of TBL allows better engagement and participation, which is conducive to more productive learning [17].

As a continuation of our previous studies, we are now investigating how the degree of preparation for each individual TBL relates to student course performance in an ID course for medical students, the only course in our curriculum where students engage in one TBL exercise each week of the course, for a total of four TBL exercises. Our analysis seeks to evaluate how weekly, pre-class preparation, as assessed by iRAT scores, relates to final examination performance amongst strongly performing and struggling students. Specifically, we hypothesize that students with better performance on the course final examination will show weekly improvements in their iRAT performance. The results of this study could provide key insights into the relationship between iRAT-TBL performance and student success in undergraduate medical education.

## Methods

The ID course is a first year medical school course, which reviews microbiology and virology as they relate to infectious diseases and their treatment. In the ID course, there are four, weekly TBL exercises, which cover all material taught in the course. Additionally, a final examination of medical knowledge composed of multiple-choice, factual questions requiring recall of facts and application of knowledge from the ID course is administered at the end of the course.

Each TBL exercise takes place at the beginning of each of week of the course, with the last exercise administered just a few days before the final examination. The TBL exercise is composed of an iRAT, a gRAT, and two application questions. Grading for each TBL exercise is based on an assignment of 33.3% to the iRAT and 66.7% to the gRAT. Each TBL accounts for 3% of the final course grade. To investigate factors that contribute to successful academic performance, we studied iRAT performance in students performing in the upper, middle and lower 33rd percentile on the final examination. These studies were approved by the Rowan Institutional Review Board (IRB).

Mean averages and standard deviations for iRAT and final examination scores amongst the various study subgroups were calculated. Changes in student iRAT scores in the four TBLs were evaluated using Kruskal-Wallis One-Way Analysis of Variance (ANOVA) and Newman-Keul’s statistical methods. We also used a Pearson correlation coefficient (r) to assess the strength and direction of the linear association between final examination and iRAT scores. For all analyses, *p* values < 0.05 were considered statistically significant and p values < 0.01 were considered highly significant. All data were analyzed using IBM SPSS 24 (Armonk, NY).

## Results

Data for this study were collected from three consecutive cohorts (Table 1), whose enrollment was 79, 86 and 95 students for years 1, 2, and 3, respectively. Overall mean average final examination and iRAT scores for the full class and students performing in the upper, middle and lower 33rd percentiles on final examination were calculated (Table 1). Pearson correlation coefficients (r) were also determined.

**Table 1:** Analysis of final examination and iRAT scores in an Infectious Disease Course for the full class and students performing in the upper, middle, and lower 33rd percentiles on the final examination

**Year 1 (*n*** **= 79)**							
	**Exam**	**iRAT Overall**	***r***	**iRAT**	**iRAT**	**IRAT**	**iRAT**
				**week 1**	**week 2**	**week 3**	**week 4**
Full Class							
	86.56 (6.87)	7.74 (1.66)	0.2674**	7.23 (1.69)	8.39 (1.54)**	7.08 (1.78)	8.28 (1.14)**
Upper 33%							
	92.75 (1.46)	8.10 (1.41)	0.0211	7.42 (1.79)	8.88 (1.07)**	7.69 (1.19)	8.38 (1.06)*
Middle 33%							
	88.12 (1.62)	7.74 (1.56)	0.1276	7.26 (1.43)	8.56 (1.21)**	6.89 (1.69)	8.26 (1.29)*
Lower 33%							
	78.76 (6.06)	7.39 (1.91)	0.3552**	7.00 (1.85)	7.73 (2.01)	6.65 (2.21)	8.19 (1.09)*
**Year 2 (*n*** **= 86)**							
	**Exam**	**iRAT Overall**	***r***	**iRAT**	**iRAT**	**IRAT**	**iRAT**
				**week 1**	**week 2**	**week 3**	**week 4**
Full Class							
	83.40 (7.71)	7.16 (1.83)	0.3289**	6.21 (1.93)	7.37 (1.74)**	6.74 (1.67)	8.32 (1.21)**
Upper 33%							
	91.76 (2.37)	7.82 (1.66)	0.2236*	6.96 (1.84)	8.00 (1.59)*	7.61 (1.42)	8.71 (1.30)**
Middle 33%							
	84.03 (2.36)	7.13 (1.83)	0.0511	5.79 (2.13)	7.63 (1.38)**	6.80 (1.63)	8.27 (1.08)**
Lower 33%							
	74.37 (4.25)	6.54 (1.80)	0.2518**	5.89 (1.64)	6.46 (1.91)	5.78 (1.50)	8.00 (1.18)**
**Year 3 (*n*** **= 95)**							
	**Exam**	**iRAT Overall**	***r***	**iRAT**	**iRAT**	**IRAT**	**iRAT**
				**week 1**	**week 2**	**week 3**	**week 4**
Full Class							
	85.39 (6.73)	8.02 (1.59)	0.3994**	7.29 (1.55)	8.60 (1.21)**	7.46 (1.84)	8.76 (1.08)**
Upper 33%							
	92.42 (2.16)	8.70 (1.29)	0.0957	7.71 (1.39)	9.32 (0.79)**	8.48 (1.29)*	9.29 (0.90)**
Middle 33%							
	86.22 (1.84)	8.08 (1.45)	0.0703	7.16 (1.53)	8.71 (1.07)**	7.71 (1.57)	8.74 (0.93)**
Lower 33%							
	77.55 (3.94)	7.31 (1.69)	0.2864**	7.00 (1.69)	7.77 (1.20)	6.19 (1.87)	8.26 (1.18)**

Data is presented as Average (Standard Deviation)

*r* = Pearson correlation coefficient comparing final examination and iRAT scores; * p < 0.05, **p < 0.01

Statistically significant differences as compared to week 1 iRAT scores are presented as *p < 0.05 and **p < 0.01

We found a positive correlation between final examination and iRAT scores for the full class in each year of this study (0.2674, *p* < 0.01, 0.3289 p < 0.01, 0.3994 p < 0.01 for years 1, 2, and 3, respectively). We found that this correlation was stronger for students performing in the lower 33rd percentile as compared to students in the upper or middle 33rd percentiles in every year of the study (Table 1). Indeed, in students performing in the upper and middle 33rd percentiles, we only found a correlation between final examination and iRAT scores in year 2 of the study (0.2236, *p* < 0.05 for the upper 33rd percentile) with no statistically significant correlation between final examination and iRAT scores in these groups in years 1 and 3.

Mean average iRAT scores for each of the four weekly TBL exercises were calculated and were compared to iRAT performance in the first week (Table 1). We detected significant increases between the first week iRAT scores and some of the iRAT scores from the second, third, and fourth week amongst students performing in the upper and middle 33rd percentiles. In students performing in the lower 33rd percentile, we only detected significant improvement between iRAT scores from the first and fourth week of the course, suggesting that their iRAT performance only improved in the final week of the course. This finding was consistent in all years of the study.

## Discussion

In this study, we provide a detailed examination of iRAT scores as they relate to final examination performance in an ID course at our institution. We found that: (1) final examination grades are most strongly correlated with iRAT scores in students performing in the lower 33rd percentile on the final examination; and (2) more consistent improvement in weekly iRAT scores was seen in students performing in the upper and middle 33rd percentiles as compared to students performing in the lower 33rd percentile, which only showed improved iRAT scores in the last TBL exercise that takes place a few days before the final examination.

As described above, iRAT scores reflect pre-class preparation, or Phase 1 of the TBL exercise [13]. Accordingly, a recent study suggests that medical students with better preparation prior to TBL, assessed by significantly more downloading of TBL-required pre-class materials, performed significantly better on graded iRAT exercises than their classmates, even after controlling for cohort effects [18]. In our ID course, students use self-directed learning time to review lecture and laboratory content in preparation for TBL exercises. Our results show that students who will ultimately struggle with mastery of course content, as assessed by the final examination, can be identified early in the course based on iRAT scores. The poor correlation between iRAT scores and final examination grades in the upper and middle 33rd percentile indicates that the ability of iRAT scores to predict final examination scores in these groups is limited. However, these students likely benefit from the preparation necessary for TBL exercises and, importantly, can provide support to struggling students in the mastery of course content during the gRAT exercise.

We also found that weekly iRAT scores consistently improve in students in the upper and middle 33rd percentiles, while students in the lower 33rd percentile only show improvement in the last TBL exercise, a few days prior to the final examination. For instance, in year 1, students in the upper 33rd percentile improved their iRAT scores from 7.42(1.79) in the first week to 8.88(1.07) *p* < 0.01 and 8.38(1.06) *p* < 0.05 in weeks two and four. Students participating in the ID course have participated of several TBL exercises prior to this course; therefore we do not believe that our data reflects adaptation of student behavior to this learning activity. The pre-class assignments are clearly identified in the course syllabus and orientation as well as in communications provided weekly prior to each TBL. An alternative explanation is that students that perform better in the course improve their pre-class preparation each week because they study more consistently and therefore better achieve the self-directed learning habits necessary for mastery of course content. On the other hand, students that perform more poorly in the course only show improved iRAT scores in the final week, a time where they are likely attempting to master all of the course content in preparation for the final examination. Of note, in some academic years we did not detect significant (*p* > 0.05) differences in iRAT scores between the first and third weeks, a phenomenon that could be explained by final examinations in other first year courses, which take place during that week.

Finally, the results presented in this manuscript are supported by the validity and reliability of ID course final examination. The office of assessment at our institution analyzes all summative assessments for various forms of validity and reliability in conjunction with course directors. As such, each examination question is linked to individual educational sessions ensuring the assessment tool has equal representation across sessions. Additionally, educational session goals and objectives are compared to question content to ensure content validity. An examination review committee, comprised of faculty members with various areas of expertise and the office of assessment, review psychometrics related to each examination question annually to look for poorly performing questions and evaluate for potential confounding variables which can affect question reliability over time. This committee is also tasked with ensuring construct validity. The examination review committee reviews thousands of questions with virtually the same group members for multiple academic years to ensure inter-rater reliability. The office of assessment also compares student written examination performance with other national test performance standards to look for relationships between internal assessment performance and national assessment tool performance. This office also conducts a detailed question item analysis over time to ensure that examinations questions are meeting internal reliability standards.

## Conclusions

Overall, this study shows that consistent review of course content, as encouraged by weekly TBL exercises, is correlated with successful course mastery in undergraduate medical education. We initially hypothesized that students with higher performance on the course final examination would show weekly improvements in their iRAT performance, likely reflecting consistent review of course materials. Our data suggest that while better performers in the course show improved iRAT performance each week, poorly performing students do not show this improvement, possibly due to failure to improve preparation for TBL exercises in the first three weeks of the course. Poorer preparation likely interferes with the efficacy of this learning method for these students and potentially their TBL teammates. As such, changes in incentives for preparation for TBL exercises could provide more tangible rewards for pre-class preparation, in particular for these students. Our study also shows that poor performance in TBL may identify a population of students that will perform poorly on the final examination. These students may need additional interventions to improve time management for self-directed learning [19] and coping skills to address the stresses inherent to medical education [20].

## Abbreviations

ANOVA: Analysis of variance
gRAT: group readiness assurance test
ID course: infectious diseases course
iRAT: individual readiness assurance test
r: Pearson correlation coefficient
TBL: Team-based learning
